# Evaluation of the cytotoxicity and antibacterial activity of nano-selenium prepared via gamma irradiation against cancer cell lines and bacterial species

**DOI:** 10.1038/s41598-024-69730-8

**Published:** 2024-09-03

**Authors:** M. Salah, Nesreen A. S. Elkabbany, Abir M. Partila

**Affiliations:** 1 Radiation Research of Polymer Chemistry Department, National Center for Radiation Research and Technology (NCRRT), Egyptian Atomic Energy Authority (EAEA), Nasr City, Cairo, Egypt; 2 Regional Center for Mycology and Biotechnology (RCMB), Al-Azhar University, Cairo, Egypt; 3 Radiation Microbiology Department, National Center for Radiation Research and Technology (NCRRT), Egyptian Atomic Energy Authority (EAEA), Nasr City, Cairo, Egypt

**Keywords:** PVP SeNPs, Gamma irradiation, Minimum inhibitory concentration, SEM, Carcinoma cell line, ESR, TEM, Genetics, Microbiology, Chemistry

## Abstract

A recent scientific investigation has shown promising results of selenium nanoparticles (SeNPs) for the anticancer and antimicrobial activities. This study aims to evaluate the effects of PVP SeNPs on bacterial strains, including *Staphylococcus aureus* (*S. aureus*), *Bacillus cereus* (*B. cereus*), *Klebsiella pneumoniae* (*K. pneumoniae*), *Escherichia coli* (*E. coli*), and *Pseudomonas aeruginosa* (*P. aeruginosa*). Also, its antitumor activity against the MRC-5 carcinoma cell line. SeNPs were prepared via gamma irradiation using PVP as a capping agent, and their size and morphological structure were determined using HRTEM. The size of the SeNPs ranged from 36 to 66.59 nm. UV–vis spectra confirmed the formation of SeNPs, while FTIR measurement confirmed a change in the PVP structure after adding selenium nanoparticles. The highest effect was reported on HepG2 by an IC50 with a value of 8.87 µg/ml, followed by HeLa, PC3, MCF-7, and Caco2 cell lines, respectively. Furthermore, ZOI reached 36.33 ± 3.05 mm. The best value of the minimum inhibitory concentration (MIC) was 0.313 µg/ml. Scanning electron microscope (SEM) imaging against bacteria showed deformations and distortions in their structures. Transmission electron (TEM) revealed ultrastructure changes in treated bacteria because of the free radicals that made cytotoxicity which confirmed by Electron spin resonance (ESR).

## Introduction

The sufficient selenium intake was reported to be between 55 and 75 µg/day with an upper limit of $\sim$ 40 mg can be obtained from certain foods such as mushrooms, vegetables, cereals, and food additives in a tea product with several health benefits. Nano-selenium has high biological activity, better bioavailability, and low toxicity compared to organic and inorganic Se-compounds such as Se(IV) and Se(VI)^1^.

Khurana et al.^2^ reported that Nano-selenium exhibited anti-tumor and anti-cancer activity via the induction of cancer cell apoptosis with minimal side effects on normal cells. Moreover, nano-selenium has a high potential to act as an antiviral, antifungal, and antibacterial^3,4^.

PVP SeNPs have gained attention in the scientific community through investigation of their anticancer activity, in addition, a great potential of this nanomaterial has been recognized recently regarding its antimicrobial activity^5^.

Antioxidant activity is one of the most fundamental features of Nano-selenium, which can remove harmful peroxides from the body through glutathione peroxidase (GSH-Px) and protect the membrane structure of organisms from damage^1^.

PVP SeNPs had surplus bioactivities and much lower toxicity than bulk Se for usages in nutritional, antimicrobial, and anticancer applications^6^.

Cancer has become a leading cause of death worldwide^7^. Chemotherapy is one of the cancer treatments. Although effective, it was restricted by their side effects and drug resistance developed by cancer cells.

Many selenium-containing compounds have been synthesized and evaluated for their anticancer activities^8^.

PVP SeNPs are emerging as promising anticancer agents because of their high bioavailability, low toxicity than selenium compounds, and remarkable anticancer activities. Previous studies found that PVP SeNPs showed potent anticancer efficacy^9^ which could be significantly enhanced by conjugating with targeting molecules such as RGD peptide^10^, folic acid^11^, and transferrin^12^.

PVP SeNPs may also be effective drug carriers that enhance the efficacy of the loaded drugs^13^. Additionally, PVP SeNPs displayed strong synergism with radiotherapy by increasing ROS production^14^.

One of the most widely employed methods to produce red Nano-selenium is the chemical reduction of selenium salts such as selenate, selenite, and selenium dioxide in the presence of ascorbic acid as a reducing agent^1^.

Why use polymers for the preparation of selenium nanoparticles? However, Se NPs are poor in stability and easy to aggregate and residue, which will reduce their antitumor activities. In addition, unmodified SeNPs cannot selectively target tumor cells, causing side effects. Stabilizers are often used to increase the stability of nanoparticles in preparation, storage, and application. Common nanoparticle stabilizers include polyvinyl pyrrolidone (PVP)^15^.

It was suggested that the presence of nanomaterials may lead to the formation of ROS. In addition, the formation of ROS in the cell can induce toxicity^16^.

Electron spin resonance (ESR) (electron paramagnetic resonance or EPR) is a powerful technique for studying chemical species with one or more unpaired electrons. ESR spectroscopy has become a direct and potent (robust-Rev1) method for detecting free radicals that are chemically generated or formed in biological systems, and nanotoxicology, this technique has been employed for detecting ROS^17^.

Transmission electron microscope (TEM) is an important technique to confirmed the ultra-structures changes in treated bacteria^18^.

## Materials and methods

### materials

#### Chemicals

Poly (vinylpyrrolidone) (PVP), M.Wt 40000, Universal Fine Chemicals PVT. LTD. Acetic acid glacial; M.Wt. 60.05, Loba Chemie. Sodium Selenite (Anhydrous) 98%, Loba Chemie. Glycerine, El Gomhouria Co., Egypt.—Tryptone was purchased from Oxoid (England), and agar and yeast extract were purchased from Oxoid, United Kingdom (UK). Dimethyl sulfoxide (DMSO), crystal violet, and trypan blue dye were purchased from Sigma (St. Louis, Mo., USA). Fetal Bovine serum, DMEM, RPMI-1640, HEPES buffer solution, L-glutamine, gentamycin, and 0.25% Trypsin–EDTA were purchased from Lonza. Crystal violet stain (1%): It is composed of 0.5% (w/v) crystal violet and 50% methanol then made up to volume with distilled H2O and filtered through a Whatman No.1 filter paper.

#### Evaluation of cytotoxic effects of certain chemical compounds (Cell lines)

*Mammalian cell lines*. HepG-2 cells (human Hepatocellular cancer cell line), MCF-7 cells (human Breast cancer cell line), CACO2 (intestinal carcinoma cells), HELA cells (human cervical cancer cell line), and PC-3 (prostate carcinoma cells) were obtained from the VACSERA Tissue Culture Unit), the American Type Culture Collection (ATCC, Rockville, MD).

#### Microorganisms (MOs)

*Bacteria cultures*. Five different strains of bacteria were collected from the Italian Hospital Lab in Egypt; *E. coli*, *P. aeruginosa*, and, *K. Pneumoniae* were employed as models for (G−ve) bacteria; *S.aureus* and *B. cereus* were also used as models for (Gram+ve). The cultures were kept on L.B. agar plates (LB agar media consists of tryptone, yeast extract, agar, and sodium chloride) at 4 °C for further studies^1,19^.

ESR measurement by EPR Spectrometer, (Bruker, Germany) located in the National Center for Radiation Research and Technology (NCRRT).

Ultrastructure examination by transmission electron microscopy (TEM), the TEM, (JEOL—JEM 1010, Tokyo, Japan at 80 kV was used to indicate the ultrastructure examination of the control and exposed bacteria to SeNPs^20^.

### Methods

#### Preparation of selenium nanoparticles

Se nanoparticles were synthesized via radical polymerization in which sodium selenite was used for producing Se nanoparticles (Na_2_SeO_3_), where polyvinylpyrrolidone (PVP) acted as a stabilizer.

1 g of Na_2_SeO_3_ dissolved in 100 ml of distilled water, 2.5% of PVP was added, and the solution was kept under magnetic stirring conditions at different rpm (rotation per minute) and (70–80 °C) of temperature for 40 min. After stirring and the dissolving of PVP, 2% acetic acid and glycerin were added at the ratio (of 1:1). Finally, the radical polymerization was achieved at gamma-ray irradiation dose at 10 KGy (dose rate 1kGy/0.66 h), and the color changed from colorless to pink color which is an indication for Nano-Selenium formation. HRTEM was used to determine the shape and the size of Nano-Selenium^21^.

#### Characterization

##### HRTEM

The ability to directly examine nanoparticles (or any solid material) in real space at or near the atomic size, i.e. the scale at which they are eventually defined, is provided by high resolution transmission electron microscopy (HRTEM). The lattice or structure of very tiny crystals (crystallites) or very small crystals in larger crystals can be observed using modern HRTEM characterizations.

To display PVP/Se particle size distribution and geometry, High-resolution Transmission Electron Microscope (HRTEM) model (JEOL/JME-2100, Japan) was employed and operated at 200 kV. To image the PVP/Se nanogel on HRTEM, the sample solution was sonicated then approximately 10–20 μL of this solution was dropped on a 3 mm copper grid, drying at room temperature. The copper grid was inserted into High-resolution transmission electron microscope.

##### Scanning electron microscopy

SEM is a method for imaging surface morphology by providing information about the shape and morphology of the materials. SEM can investigate the surface morphology of nanoparticles because it detects scattered electrons from the particle's surface.

The surface topography and structural features of the prepared PVP/Se NPs nanogel after drying was examined using scanning electron microscopy (SEM) (ZEISS-EVO 15-UK). EDX was utilized to identify and investigate the elemental composition. EDX attached to scanning electron microscopy (SEM) (ZEISS-EVO 15-UK).

##### UV–Vis spectrophotometry

The prepared PVP/Se nanogel is characterized by measuring optical absorbance (A) in the wavelength range 200–800 nm using double beam spectrophotometer model JASCO 670 UV–Vis–NIR. For this measurement, the sample solution was diluted by using bi-distilled water (0.1 mL of sample/1 mL of bi-distilled water) to be used for the measurement of the absorption spectra.

##### FTIR analysis

FTIR Model Cary 630 FTIR spectrometer was used to record the FTIR analysis of original PVP, and the nanogel of PVP/Se NPs in the wavelength range 4000–400.

##### XRD analysis

XRD of the dried PVP/Se NPs was performed according to XRD spectrophotometer, Model (X’pert Pro), Manufacture (PAN analytical) equipped with X-ray tube [Cu target, 40 kV (Voltage), 30 mA (current)], the X-ray data were recorded in a range from 4 to 80°, 2θ with continuous scanning mode and scanning speed 8°/min.

##### Raman spectroscopy

SENTERRA II Raman Microscope was used to record the Raman analysis of the nanogel of PVP/Se NPs.

#### Anticancer assessment

For antitumor assays, the tumor cell lines were suspended in the medium at a cell density of 5 × 104 cells/well in Corning® 96-well tissue culture plates and then incubated for 24 h. The tested compounds were then added to 96-well plates (six replicates) to achieve eight concentrations for each compound. Six vehicle controls with media or 0.5% DMSO run for each 96-well plate as a control. After incubating for 24 h, the numbers of viable cells were determined by the MTT assay^22^. Briefly, the media removed from the 96-well plate and replaced with 100 µl of fresh culture RPMI 1640 medium without phenol red then 10 µL of the 12 mM MTT stock solution {5 mg of 3-(4,5-dimethylthiazol-2-yl)-2,5-diphenyltetrazolium bromide purchased from Sigma-Aldrich (St. Louis, MO) in 1 mL of Phosphate buffered saline} added to each well including the untreated controls. The 96-well plates were then incubated at 37 °C and 5% CO_2_ for 4 h.

An 85 µl aliquot of the media was removed from the wells, 50 µl of DMSO was added to each well, mixed thoroughly with the pipette, and incubated at 37 °C for 10 min.

Then, the optical density was measured at 590 nm with the microplate reader (Sunrise, TECAN, Inc, USA) to determine the number of viable cells, and the percentage of viability was calculated as;

$$[{\text{ODt}}/{\text{ODc}}] \times {1}00\%$$

where ODt is the mean optical density of wells treated with the tested sample and ODc is the mean optical density of untreated cells. The relation between surviving cells and drug concentration is plotted to get the survival curve of each tumor cell line after treatment with the specified compound. The 50% inhibitory concentration (IC50), the concentration required to cause toxic effects in 50% of intact cells, was estimated from graphic plots of the dose–response curve for each conc. I am using GraphPad Prism software (San Diego, CA, USA)^23^.

*Safety and selectivity index (SI)*. The effects of the tested compounds and cisplatin reference drug were measured on normal human lung fibroblast (MRC-5) cell line (obtained from the American Type Culture Collection, ATCC, Rockville, MD) as mentioned previously to produce a dose–response curve and to calculate the 50% cytotoxic concentration (CC50) using GraphPad Prism software. The SI was calculated by dividing the CC_50_ by the IC_50_ values. A SI of > 10 indicates the safety of a compound.

#### Qualitative anti-microbial characterization of nanocomposite PVP SeNPs

By Zone of Inhibition (ZOI) and confirmed by Scanning Electron microscopy (SEM) in which the disc diffusion technique was applied to determine the zone of inhibition by the ZOI assay “zones of bacterial inhibition” after exposure of bacteria to nanocomposites^24^. Sterile discs (10 mm diameter) of Whatman No. 2 filter paper were loaded by 25, 50, and 100 µL/disc of Nanocomposite PVP/Se NPs of concentration 1 g/l, and positioned on the surfaces of freshly LB agar plates inoculated with bacterial cultures, for the standardization of the prepared inoculums, they were compared using the Mcfarland scale 10^5^ CFU. Perti dishes were allowed to incubate at 37 °C for 24 h, and the zone of inhibition was found, diameters were measured accurately in triplicates, in which the inhibition zone was measured as a line passe throughout the center of the disc and subtracted from it the diagonal of the disc), and their means were reckoned^19^.

For the ‘‘Scanning Electron microscopy” (SEM) imaging, the SEM (Evo 15 Zeiss UK) was used for detecting the morphological alterations in treated bacterial cells with a fixed volume of the Nanocomposite PVP/Se NPs for 18 h of treatment and incubation at 37 °C. The image capturing tracked the structural distortions in exposed cells^19^.

#### Quantitative antimicrobial examination of nanocomposites

First, a fixed volume of Nanocomposite PVP/Se NPs diluted to 8 concentrations from 1000 to 0.156 μg/ml. All pre-cultured strains were grown on LB broth at 37 °C for 18 h, The cultured bacteria were diluted using LB broth media to obtain bacterial cell densities of approximately 5 × 10^5^ CFU/mL. Nine hundred microliters of the diluted bacterial suspension inoculated test tube containing 100 μL of serially diluted tested nano compounds. As a positive control, 100 μL of PBS was added to 900 μL of the bacterial solution, and 100 μL PBS was added to 900 μL of LB broth media as a negative control. Test tubes were incubated at 37 ± 2 °C for 18 h, with shaking. MIC is defined as the lowest concentrations in which there was no visible growth of microorganisms (modified)^25^.

In which Serial dilutions for Nanoparticles were performed for the first two dilutions 100 and 10 μg/mL then at 1:1 ratio, ranging from 10 to 0.156 μg/mL.

TEM is required to see the ultrastructure changes inside the bacterial cell and also ESR to understand the mechanism that causes these changes.

Electron Spin Resonance (ESR) was measured to detect free radicals that formed chemically in biological systems and to investigate the energy absorption spectra, by BRUKER EMX EPR Spectrometer, (Bruker, Germany), using a standard rectangular cavity of ER 4102, modulation frequency, 100 kHz, Source of the electromagnetic radiation (generator of microwave radiation, its called Klystron -X-band has frequency 9.5 Giga Hertz and wavelength is about 3 cm), Appropriate detection system (cavity sensors) to monitor the amount of radiation absorbed by the sample, also electromagnet to give a magnetic field to concentrate the electromagnetic radiation on the sample, Waveguide to transfer the microwave radiation from klystron to the sample, and quartz tubes have an inner diameter about 3 or 5 mm are generally used to contain powder of bacterial sample which was grown for 24 h incubation along with nanoparticles, then dried^26^.

##### Preparation and examination of bacteria using electron microscopy TEM

The ultrathin sections and the morphological alternations of the prepared bacterial samples by TEM (*P. aeruginosa*), treated with (0.156 μg/mL SeNPs) compared with control cells were prepared as follows: bacterial cells were collected by centrifugation at 4000 rpm for 10 min from 24-h-old cultures grown on nutrient broth media, the samples were fixed in 3% glutaraldehyde, rinsed in phosphate buffer, and post-fixed in potassium permanganate solution for 5 min at room temperature. The samples were dehydrated in an ethanol series ranging from 10 to 90% for 15 min in each alcohol dilution and finally with absolute ethanol for 30 min. Samples were infiltrated with epoxy resin and acetone through a graded series until finally in pure resin. Ultrathin sections were collected on copper grids. Sections were then double stained in uranyl acetate and then in lead citrate. Stained sections were observed using transmission electron microscopy (JEOL-JEM 1010) at 80 kV at the Regional Center for Mycology and Biotechnology (RCMB), Al-Azhar University^27^.

### Statistical analysis

The data were expressed as mean ± S.D. STATA statistical analysis package was used for the dose–response curve drawing in order to IC_50_ calculations.

## Results

### Characterization

#### HRTEM measurement (first run)

**Figure 1:**
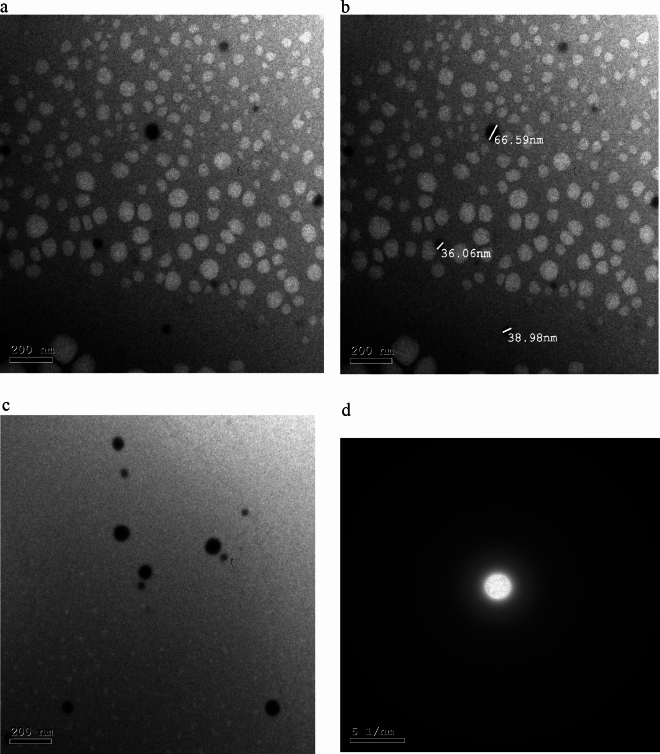
HRTEM images of nano-selenium stabilized with PVP, (**a**, **b**, and **c**) selenium nanoparticles and their corresponding size, (**d**) SAED pattern of prepared selenium/PVP nanocomposite.

Figure 1a, b, and c shows HRTEM images of synthesized nano-selenium and the size dispersion at different locations. These images depict that the shape of nano-selenium is spherical. Also, no aggregations were observed in these images. The particles diameters are in the range (36–66.59) nm.

#### Elemental composition analysis (EDS)

Energy-dispersive X-ray analysis (EDAX) is a technique used for measuring the nanoparticles by HRTEM. In this technique, the nanoparticles are analyzed by activation using an EDS X-ray spectrophotometer, which is generally present in modern HRTEM and SEM. The method relies on the generation of characteristic X-rays that reveal the identity of the elements present in the sample.

#### Shows the weight and atomic (%) composition of Se NPs sample by EDS

**Figure 2:**
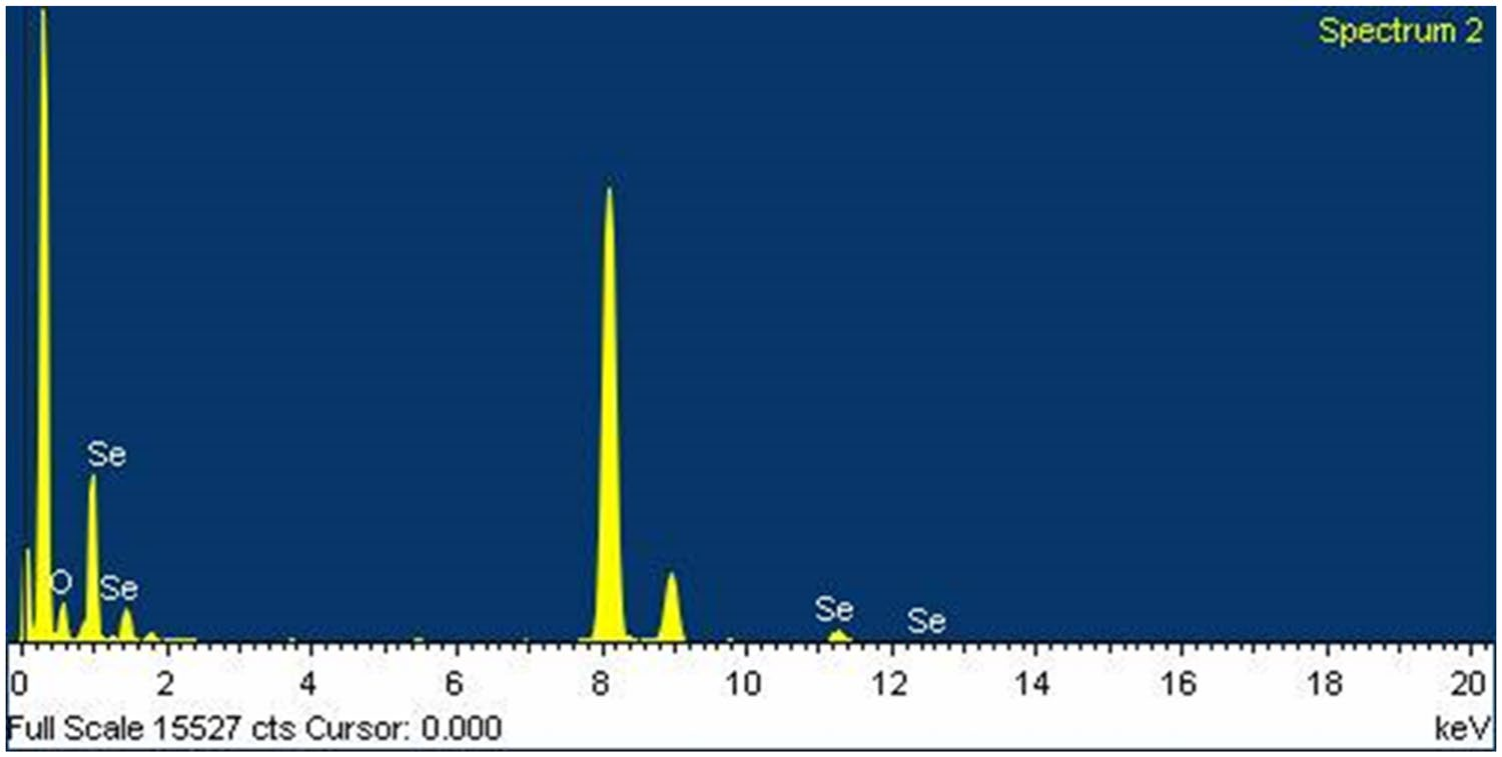
Displays the EDS pattern of Selenium (Se) nanoparticles.

EDX analysis of the prepared Se NPs (Fig. 2) exhibited the characteristic Se absorption peaks at 1, 1.37, 11.22, and 12.49 keV. Moreover, EDX analysis revealed the presence of the absorption peak of oxygen (O) around 0.45 keV. These peaks are evidence for the formation of selenium nanoparticles. As shown in Table 1, the weight percentage and atomicity of Se and oxygen (O) were found to be (55.27, 20.03); (44.73, 79.97), respectively.

**Table 1:** Shows the weight and atomic (%) composition of Se NPs sample by EDS.

Element	Weight	Atomic
O K	44.73	79.97
Se L	55.27	20.03
Totals	100	100

#### HRTEM imaging (second run)

**Figure 3:**
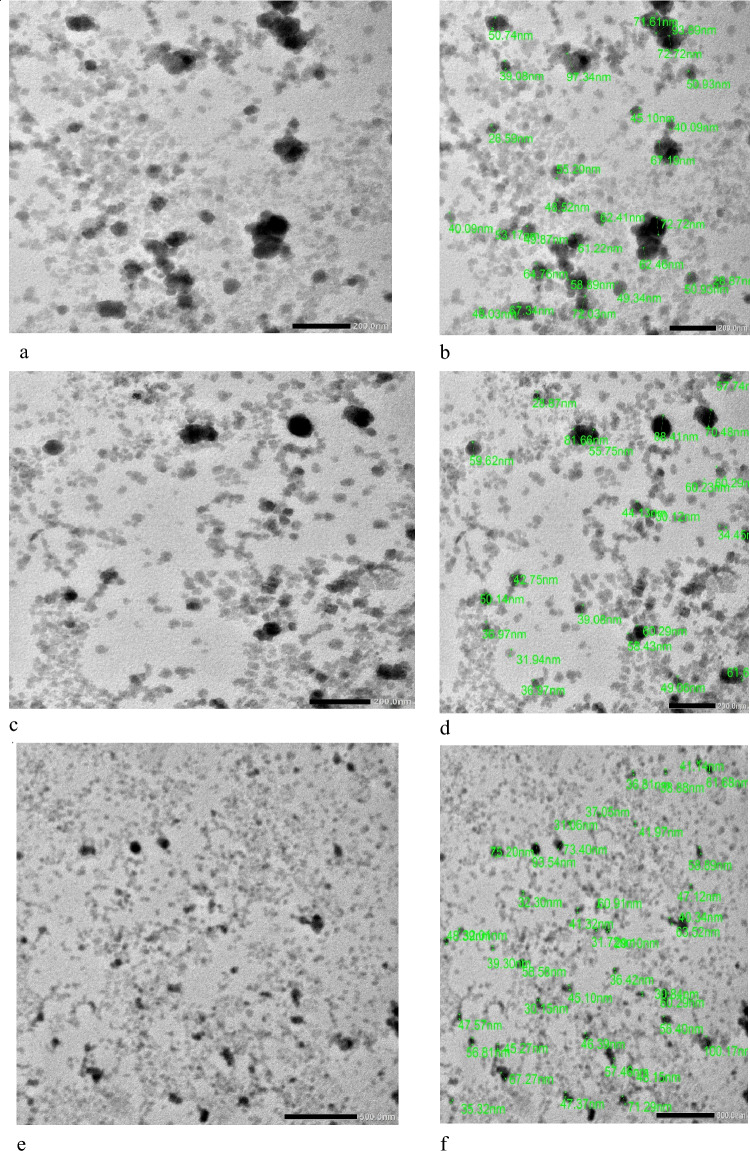

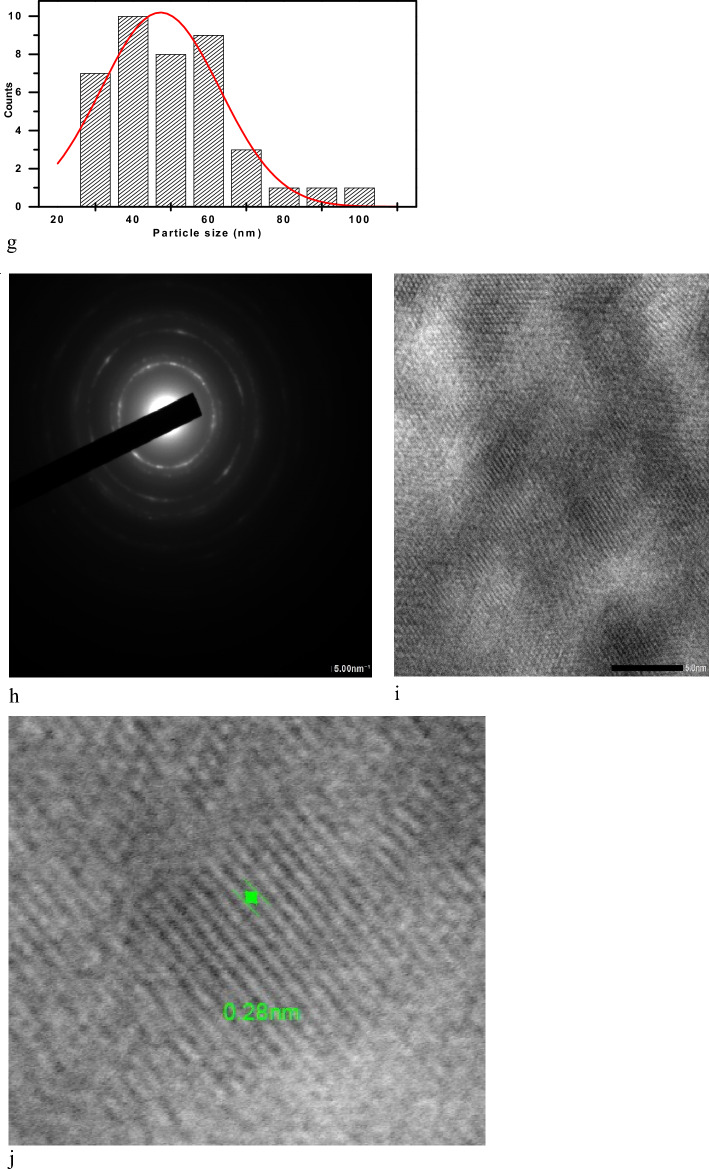
HRTEM images of selenium nanoparticles (**a**, **c**, and **e**) at different scale bar magnifications, images (**b**, **d**, and **f**) size distribution of Se NPs, (**g**) size distribution histogram of Se NPs, (**h**) SAED pattern of Se NPs, (**i**) lattice fringes of Se NPs, (**j**) the interplanar spacing which corroborates the d-spacing value of 0.28 nm.

High-resolution TEM (HR-TEM) is a phase-contrast imaging technique, enabling the acquisition of images with a near-atomic resolution, allowing investigation of the crystallinity, lattice planes, crystal phases, and defects (Fig. 3).

#### Surface morphology (SEM) and EDX spectroscopy

**Figure 4:**
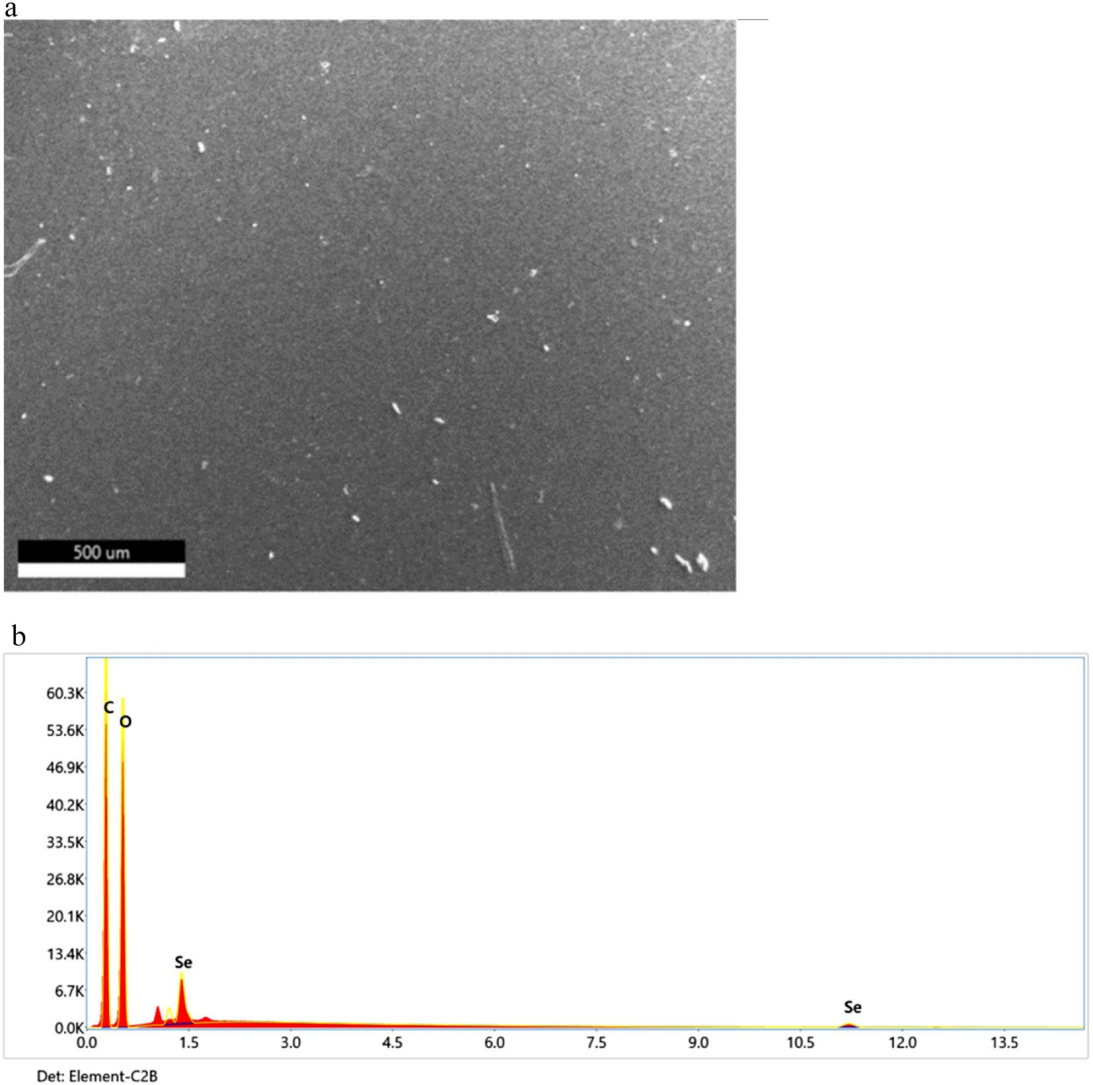
(**a**) Shows SEM image of PVP/Se NPs at the surface mode. (**b**) EDX spectra of PVP/Se NPs.

Figure 4 displays the surface morphology and elemental analysis of the produced Se NPs. The elemental structure was examined using EDX spectroscopy, which was also used to verify the generated samples.

Similarly, the SEM image of the Se NPs that spread across the PVP's surface as brilliant particles is displayed in Fig. 4a. Furthermore, a homogeneous distribution of Se NPs was observed on the surface.

With EDX (Fig. 4b), distinctive X-rays are identified and connected, based on their wavelength and intensity, to the existence of particular elements and their concentration in a sample. Moreover, Table 2 summarized the weight and atomic (%) composition of Se NPs sample obtained from EDX.

**Table 2:** Shows the weight and atomic (%) composition of Se NPs sample by EDX.

Element	Weight %	Atomic %
C K	48.53	56.75
O K	48.72	42.76
SeK	2.75	0.49

#### SEM/EDX elemental mapping analysis

**Figure 5:**
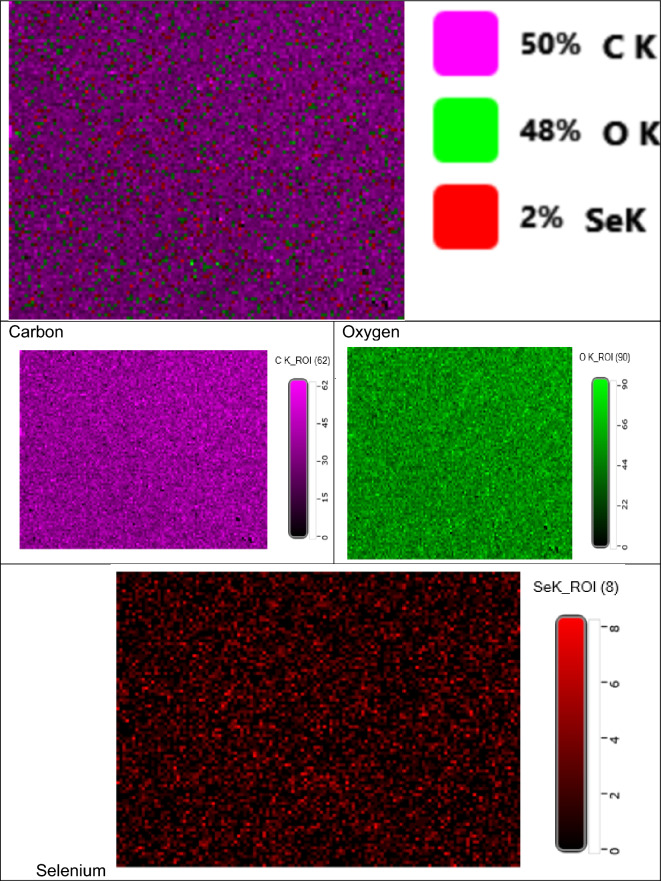
Illustrates elemental mapping of PVP/Se NPs sample.

SEM–EDS mapping combines the capability of X-ray spectroscopy with the spatial resolution of an advanced electron microscope. Figure 5 illustrates elemental mapping of PVP/Se NPs sample.

#### Crystallinity degree characterization via XRD

**Figure 6:**
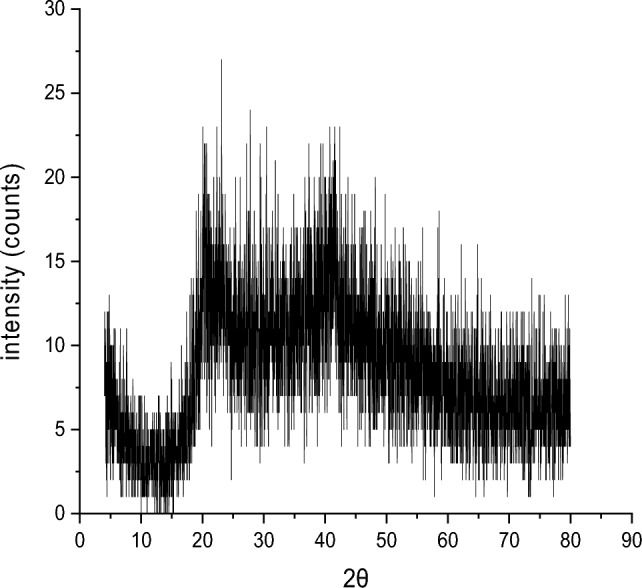
XRD pattern of PVP/Se NPs.

XRD analysis was used to examine the crystal structure and phase of the produced NPs. The synthetic Se NPs' XRD pattern is shown in Fig. 6. It is distinctly displayed in the pattern that there are no characteristic peaks for the starting precursors sodium selenite.

#### UV–Vis spectroscopy analysis and stability monitoring

The maximum absorbance and the corresponding wavelength for the sample were observed in UV/Vis spectroscopy, which was used to monitor the stability of nanofluids. UV–vis spectroscopy is used to characterize nanoparticles by detecting the surface plasmon resonance (SPR) band, which indicates the formation of nanoparticles.

**Figure 7:**
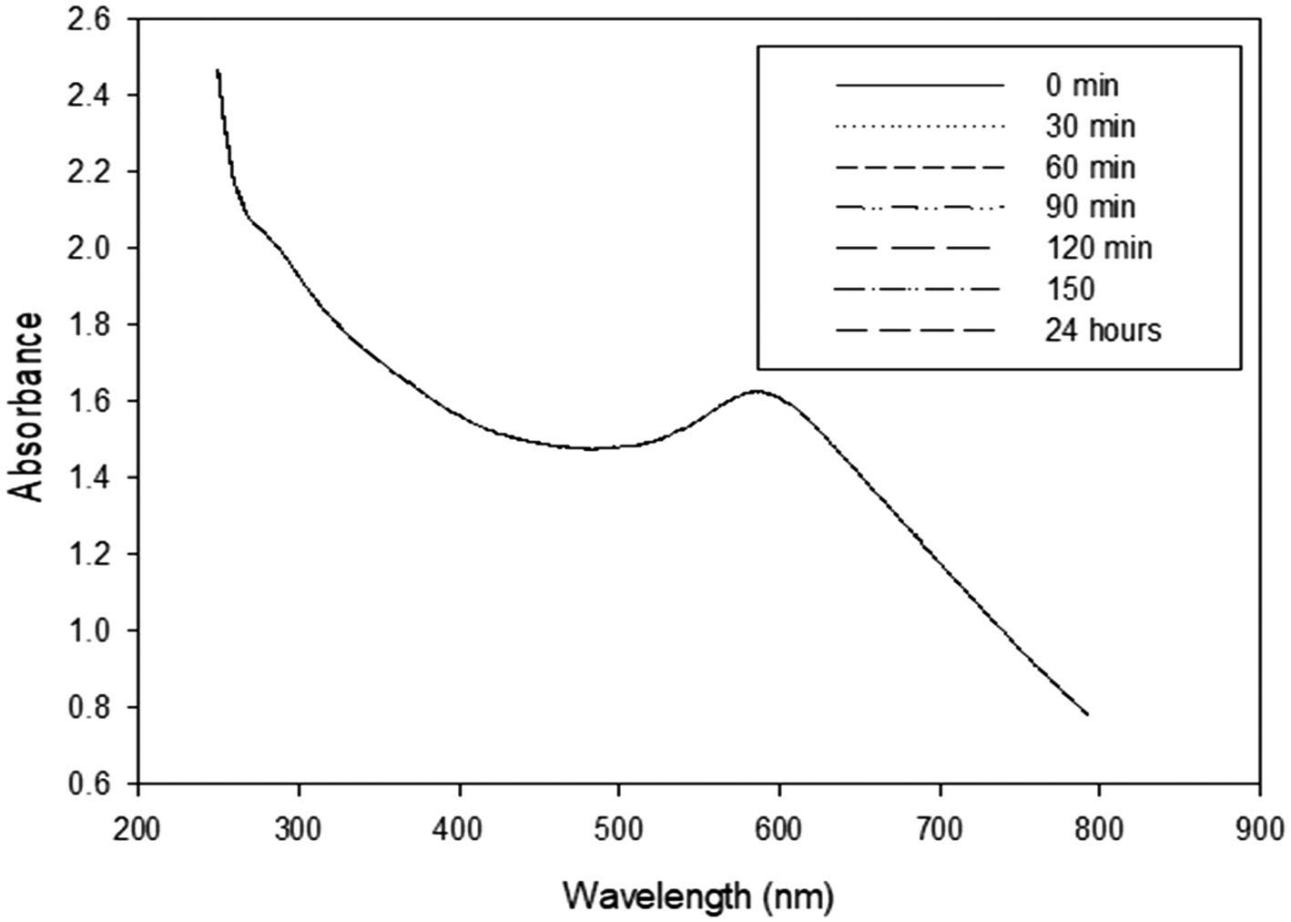
UV spectrum of the prepared nano-selenium plotted as absorbance and measured at different time intervals.

Ultraviolet and visible (UV–Vis) absorption spectroscopy is the technique by which we measure the attenuation of light that passes through an under-consideration sample or after reflection from the sample. Both parts (UV and Vis) of light are energetic and can excite electrons to higher energy levels. The reduction of selenium ions to selenium nanoparticles was evidenced by the visual color change from yellow to reddish due to the excitation of surface Plasmon vibrations in Se NPs. Such a color change of Se nanoparticle dispersion manifested by the UV–visible spectra. In this regard, the UV spectrum was plotted as absorbance (Fig. 7).

UV–Vis spectroscopy was utilized to better analyze Se-NPs. The surface plasmon resonances of selenium nanoparticles are the source of a moderate absorption band at 280 nm and a strong absorption band with a maximum at 580 nm that were discovered, as shown in Fig. 7.

#### FTIR spectroscopy

Fourier transform infrared spectroscopy (FTIR) was used extensively to characterize the quantitative analysis of polymer blends, determine their compatibility via intermolecular hydrogen bonding, and investigate their degradation processes. FTIR spectroscopy has been applied for blending studies because the physical properties of polymer blends are affected by the structures of the molecular chains.

The Table 3 summarizes the obtained IR bands and their functional group description which is explored in detail below.

**Table 3:** Declares the obtained IR bands and its functional group description.

IR band	Functional group description
3433 and 1368 cm^−1^	O–H stretching vibration
1648 cm^−1^	C=O stretching vibration
1427 cm^−1^	The methylene group (CH_2_) bending vibration
2945 cm^−1^	The asymmetric stretching vibration of the CH group
2894 cm^−1^	C–H stretching vibrations
1276 and 1074 cm^−1^	C–N bond
2170.8 cm^−1^	C–C=–C–C=–CH
2024.96 cm^−1^	C=C and C=N stretching

**Figure 8:**
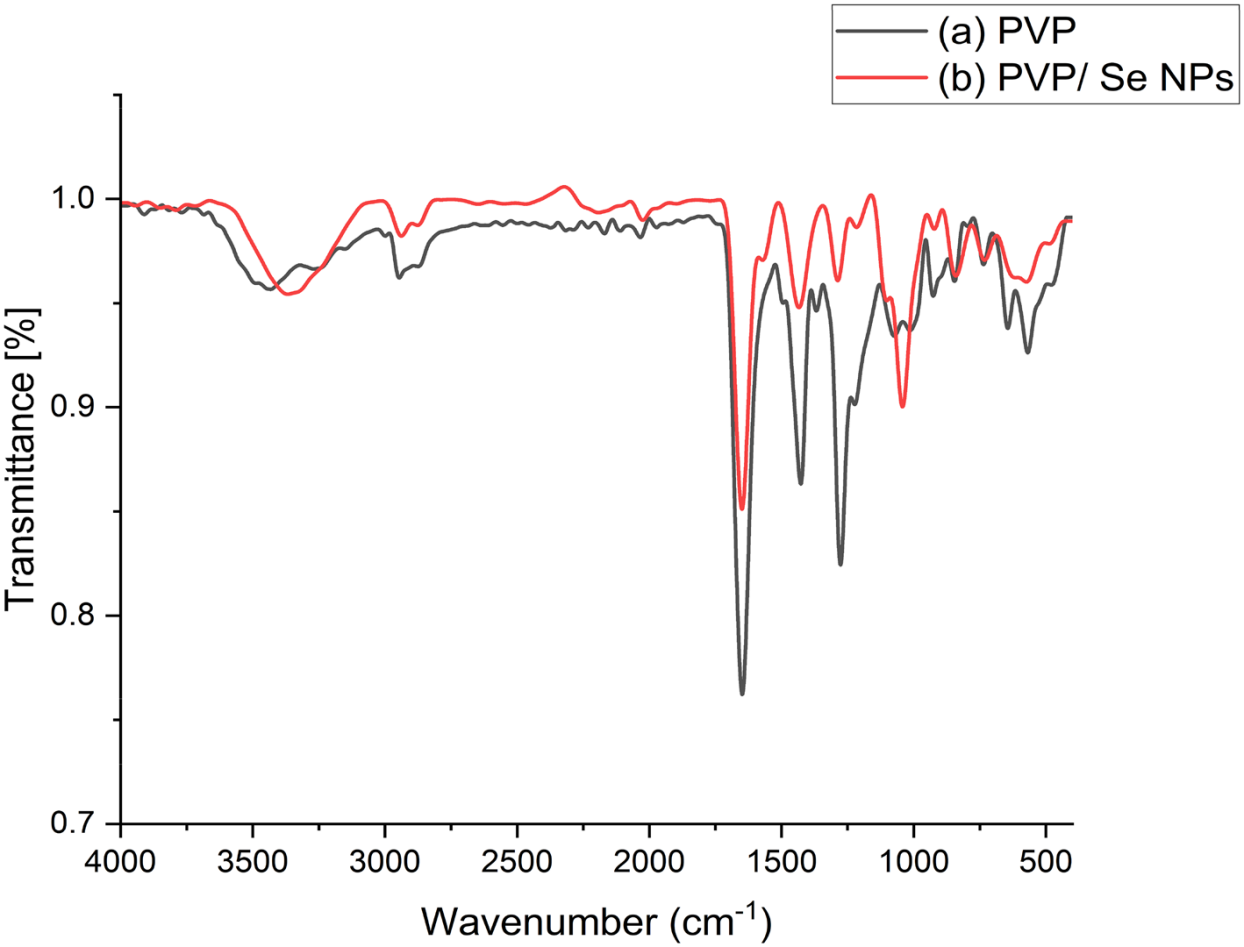
FTIR spectra of (**a**) PVP and (**b**) PVP/Se NPs.

Figure 8a shows the characteristic peaks related to the functional groups of the polyvinylpyrrolidone (PVP); the peaks at approximately 3433 and 1368 cm^−1^ refer to the O–H stretching vibration, the band at 1648 cm^−1^ is associated with the C=O stretching vibration, and the bands at 1427 cm^−1^ to the methylene group (CH_2_) bending vibration, while the peak at 2945 cm^−1^ is related to the asymmetric stretching vibration of the CH group. In addition, the signals related to the C–N bond at 1276 and 1074 cm^−1^.

As noted in Fig. 8b, there are differences after the preparation of selenium nanoparticles using PVP solution combined with the additive’s acetic acid and glycerol; the displacement of the peak centered at 3433–3368 cm^−1^. Also, the intensity increased for the same peak. Moreover, the FTIR spectra showed differences in the intensity and broadness of the peaks due to the chemical interaction between Se NPs and the polymeric network, the other most significant differences are as follows; The observed band at 2170.8 cm^−1^ and the noticed peak at 2024.96 cm^−1^. Furthermore, it was found the decreasing intensity of FT-IR bands at 1434 and 1287 cm^−1^ and disappearing the peak of 1368 cm^−1^, also.

#### Raman spectroscopy

**Figure 9:**
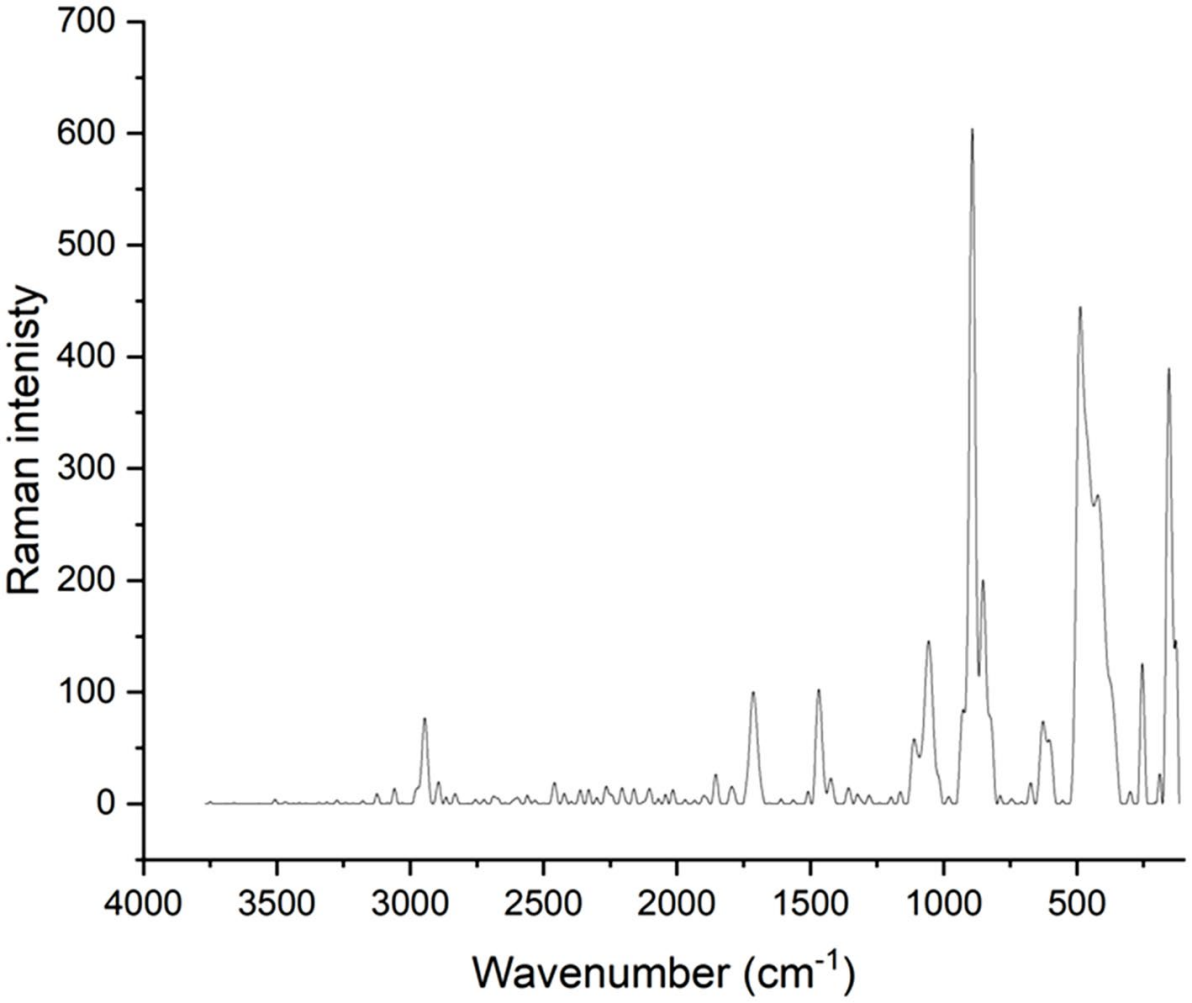
Displays Raman spectra of PVP/Se NPs.

It is well known that variations in the several allotropic modifications and crystallinity of selenium in SeNPs may be detected by Raman spectroscopy as illustrated in Fig. 9 and that the Raman spectra can be interpreted using empirical characteristic frequencies.

### Antitumor assessment

**Figure 10:**
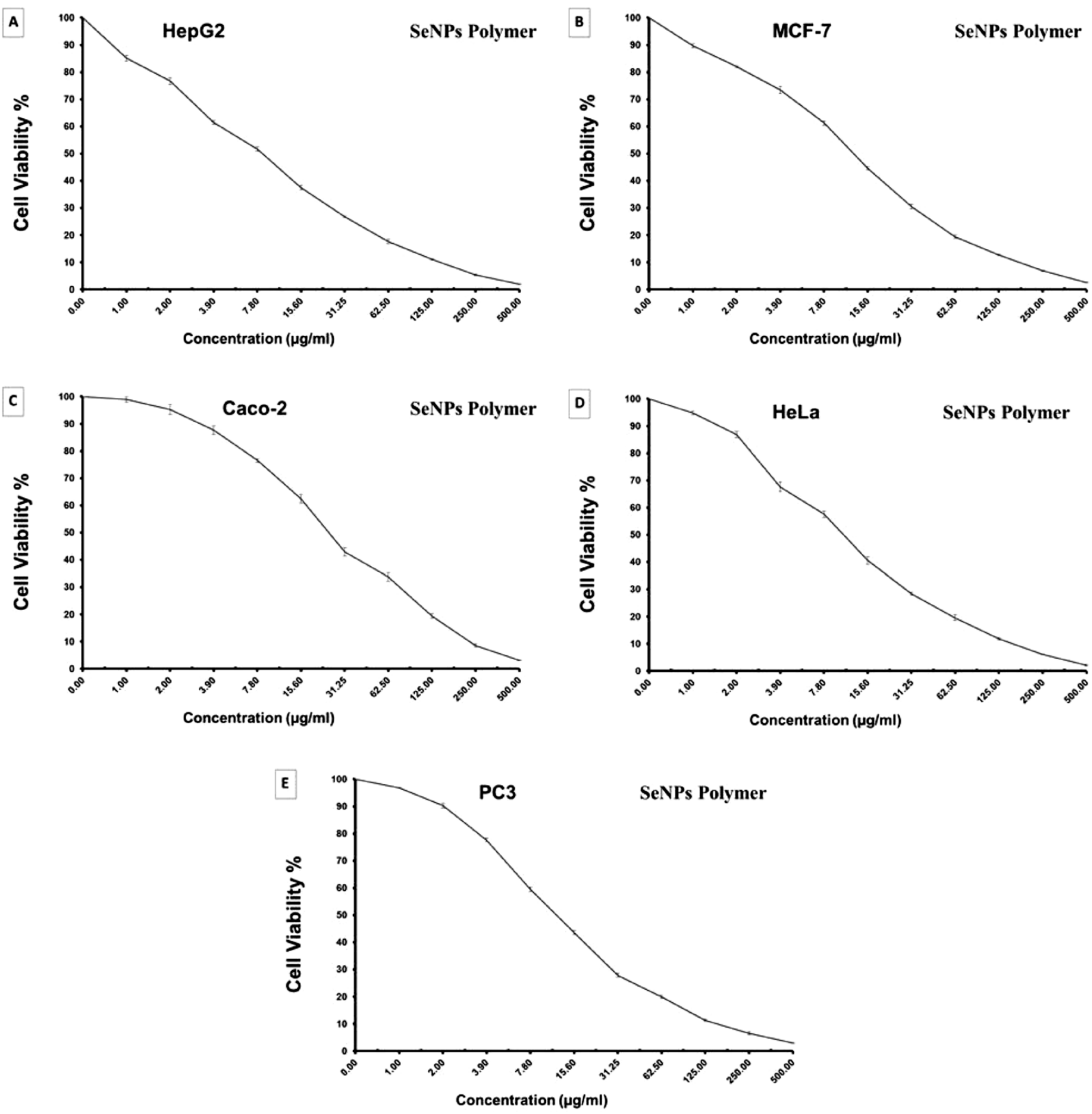
The dose response curve showing the in vitro inhibitory activity of the synthesized PVP SeNPs against the five tested carcinoma cell lines. (**A**) liver carcinoma (HepG2) cell line; (**B**) breast carcinoma (MCF-7) cell line; (C) colorectal carcinoma (Caco2) cell line; (**D**) cervix carcinoma (HeLa) cell line; (**E**) prostate carcinoma (PC3) cell line. The data are expressed as mean ± S.D.

The growth inhibitory activity of the synthesized Nanocomposite PVP SeNPs was investigated in vitro under the same conditions, using colorimetric MTT assay. A dose–response curve was plotted (Fig. 10), and the concentration required to eradicate 50% of the cell population (IC50). The results revealed that all the tested compounds showed variation in inhibitory activity to the five tested carcinoma cell lines in a concentration-dependent manner.

**Figure 11:**
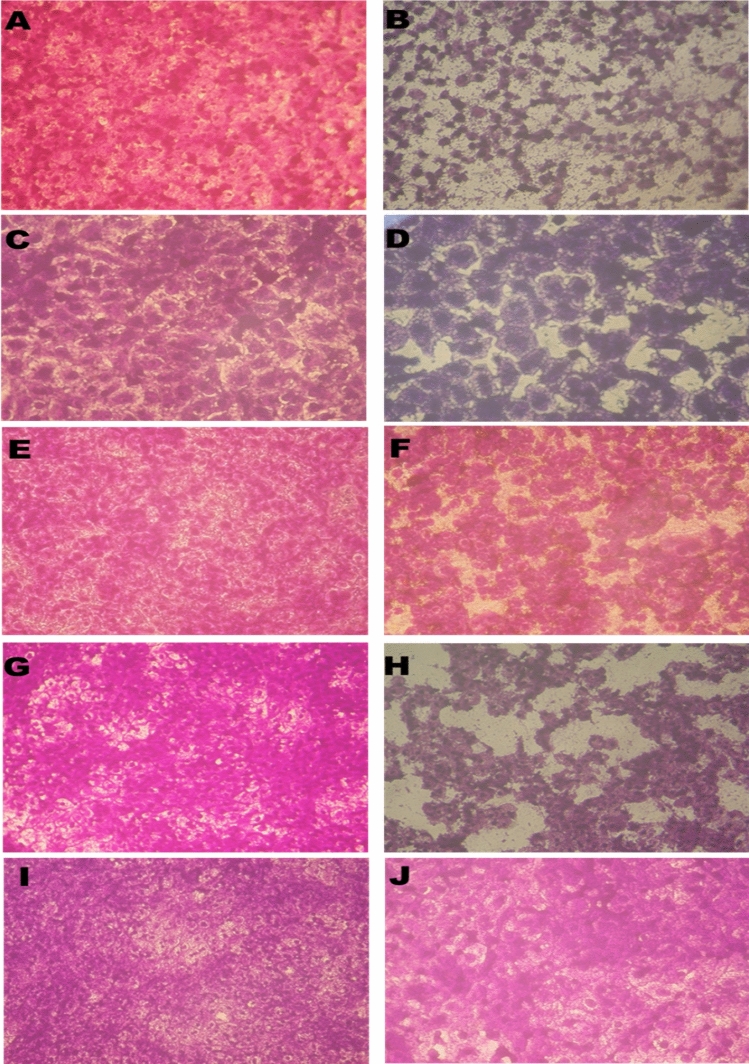
The morphological cell changes showing inhibitory activity of the synthesized PVP SeNPs against liver carcinoma (HepG2) cell line (**A**) control non-treated and (**B**) treated; breast carcinoma (MCF-7) cell line (**C**) control non-treated and (**D**) treated; colorectal carcinoma (Caco2) cell line (**E**) control non-treated and (**F**) treated; cervix carcinoma (HeLa) cell line (**G**) control non-treated and (**H**) treated; prostate carcinoma (PC3) cell line (**I**) control non-treated & (**J**) treated.

Moreover, the highest inhibitory activity reported against liver carcinoma (HepG2) with an IC50 value of 8.87 µg/ml, then cervix carcinoma (HeLa), prostate carcinoma (PC3), breast carcinoma (MCF-7), colorectal carcinoma (Caco2) cell lines, respectively (Figs. 10, 11 and Table 4). The effects of the tested Nanocomposite PVP SeNPs were also measured on normal human lung fibroblast (MRC-5) cell lines to produce a dose–response curve and to calculate the 50% cytotoxic concentration (CC50) as indicated in Table 4. The selectivity index (SI) was calculated by dividing the CC50 by the IC50 values.

**Table 4:** The antitumor activities of the tested selenium nano complex against carcinoma cell lines (expressed as IC_50_ values) along with selectivity index when compared against non-cancerous cells.

Tested cell line	Organ	IC50 values* (µg/ml)	SI (CC50/IC50)
HepG-2	Liver cancer	8.87 ± 0.69	12.34
MCF-7	Breast cancer	12.81 ± 1.03	8.55
Caco2	Colorectal cancer	25.19 ± 1.75	4.35
HeLa	Cervical cancer	11.24 ± 0.98	9.74
PC3	Prostate cancer	12.26 ± 0.89	8.93
MRC5	Non-cancerous lung fibroblast	109.51 ± 4.37	–

*The data are expressed in the form of mean ± standard error.

Interestingly, when the tested composite was evaluated for their toxicity against normal cells, they exhibited low toxic effects, indicating the safe use of most of them that may require further studies in vivo and pharmacologically.

Moreover, nano-Se potentiates MCF7 and Caco-2 chemo-sensitivity inhibits cancer cell bioenergetics moreover, synthesized Selenium Nanoparticles have anticancer against the MCF-7 cell line, exhibited a dose-dependent effect using the MTT assay, and the inhibitory concentration (IC50) was 50μg/ml.

The dose of 100μg/ml of Nanocomposite PVP/Se NPs-Chit was cytotoxic, but at all lower concentrations, more than 80% of cells were viable.

### Microbiological assessment

The bacterial inhibitory actions of fabricated polymers, Nanocomposite PVP/Se NPs were assessed qualitatively by ZOI measurement and quantitatively (via MIC determination) and confirmed through SEM imaging for the Gram-negative (*K. pneumonia*, *E. coli*, and *P. aeruginosa*) and Gram-positive (*S. aureus* and *B. cereus*) bacteria.

**Figure 12:**
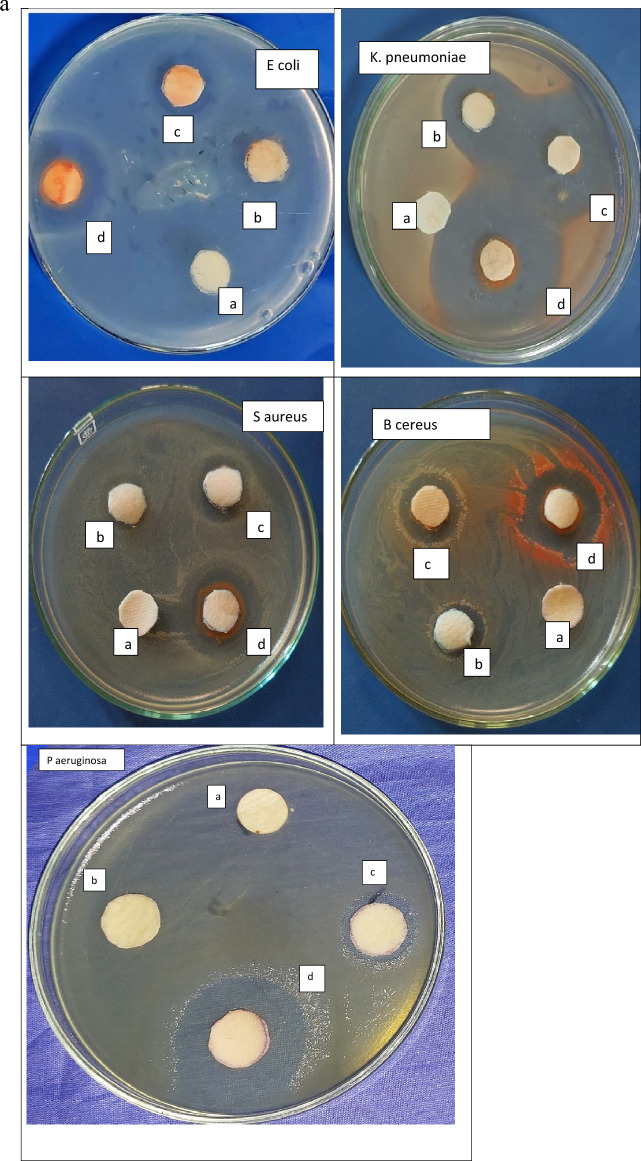

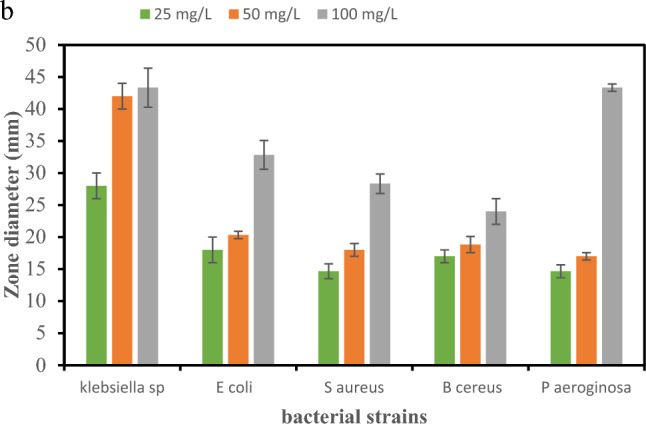
(**a**) Antibacterial inhibition zone with different Se NPs concentrations: (**A**) pure sample, (**B**) 25, (**C**) 50, and (**D**) 100 ul of Se NPs. (**b**) Antibacterial activity of PVP/Se NPs against bacterial strains. Significant difference at *p* ≤ 0.05”.

Figure 12a, b and Table 5 show the results of the antibacterial activity of PVP/Se NPs. The concentration of dispersed selenium nanoparticles affected *P. aeruginosa* and *K. pneumonia* more than the other species*.*

**Table 5:** MIC values for nanoparticles against *K. pneumoniae*, *E. coli*, and *S. aureus*, *B. cereus* bacteria and *P. aeruginosa*.

MOs	ZOI (mm)			MIC (µg/ml)
	25	50	100	
*K. pneumoniae*	21.00 ± 2.00^b^	35.00 ± 2.00^a^	36.33 ± 3.05^a^	0.313
*P. aeruginosa*	7.00 ± 1.500^c^	11.00 ± 1.00^b^	36.33 ± 3.05^a^	0.313
*E. coli*	11.00 ± 1.400^b^	13.33 ± 0.57^b^	25.83 ± 2.25^b^	0.625
*S. aureus*	7.66 ± 1.15^c^	11.00 ± 1.00^b^	21.33 ± 1.52^b^	1.252
*B. cereus*	10.00 ± 1.00^c^	11.83 ± 1.25^b^	17.00 ± 2.00^b^	1.252

^a^Highly significant.

^b^Moderate significant.

The obtained results also revealed that by increasing the Se NPs, the antibacterial activity increased.

The results of the antibacterial activity test showed that all samples containing Se NPs had an antibacterial influence against all strains. Moreover, it found that the antibacterial activity of the G -ve is better than the antibacterial activity of the G+ve.

#### Minimum inhibitory concentration (MIC)

The nanostructures were active toward both the Gram-negative and positive bacteria at a range of concentrations between 1000 and 0.157 µg/ml, with a higher impact when the PVP/Se NPs presented in the cell cultures of G−ve. The results of MIC showed a dose-dependent inhibition of bacterial growth.

In this study, the Nanocomposite PVP/Se NPs showed MIC values in Table 5 around 0.313 when cultured with *K. pneumoniae* and *P. aeruginosa*, it was 0.625 with *E.* *coli*, while 1.252 with *S. aureus* and *B. cereus*. Their values differ from those found in other works of literature, showing either a decrease or similarity to the MIC values as seen in the discussion part.

#### Scanning electron microscope (SEM)

**Figure 13:**
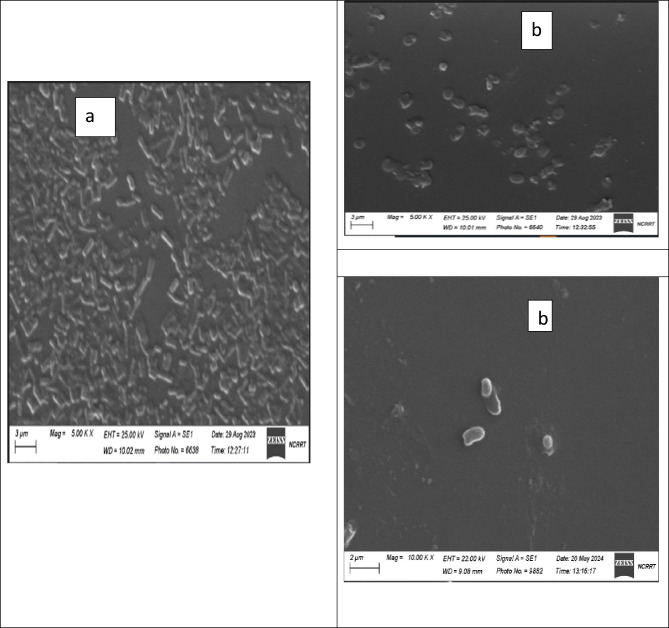

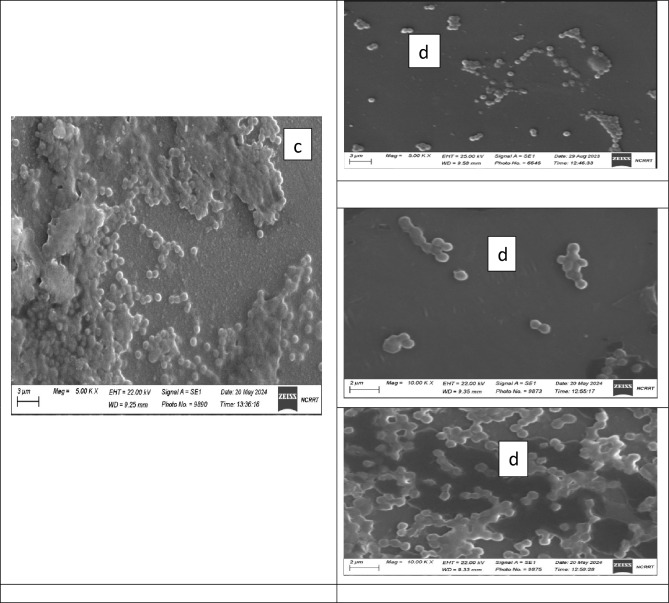

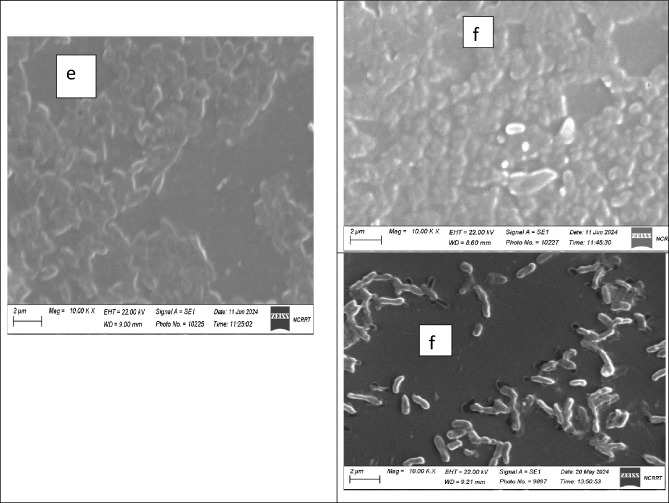
Scanning imaging of exposed *E. coli*, *S. aureus*, and *P. aeruginosa* cells to (Nanocomposite PVP/Se NPs), (**a**), (**b**) refer to *E. coli*; (**c**), (**d**) for *S. aureus*, and (**e**), (**f**) refer to *P. aeruginosa* before and after the exposure, respectively.

The antibacterial action of PVP SeNPs was elucidated through SEM imaging by exposing bacteria (*E. coli*, *P. aeruginosa*, and *S. aureus*) to the fabricated nanocomposite. At no exposure (E, P and S-control in Fig. 13), the cells’ appearance was natural, with contacted and smooth surfaces, After exposure for 18 h, some nanocomposite particles were detectable in attachment with bacterial outer membranes, the deformations, and distortions in cells was evidenced, most cells began to lose their distinctive features and their membranes entered the lysis phase and mostly lysed or exploded, and the bacterial action was more vigorous in *E. coli* and *P. aeruginosa* cells.

#### Effects of SeNPs exposure of sample on ESR spectra

**Figure 14:**
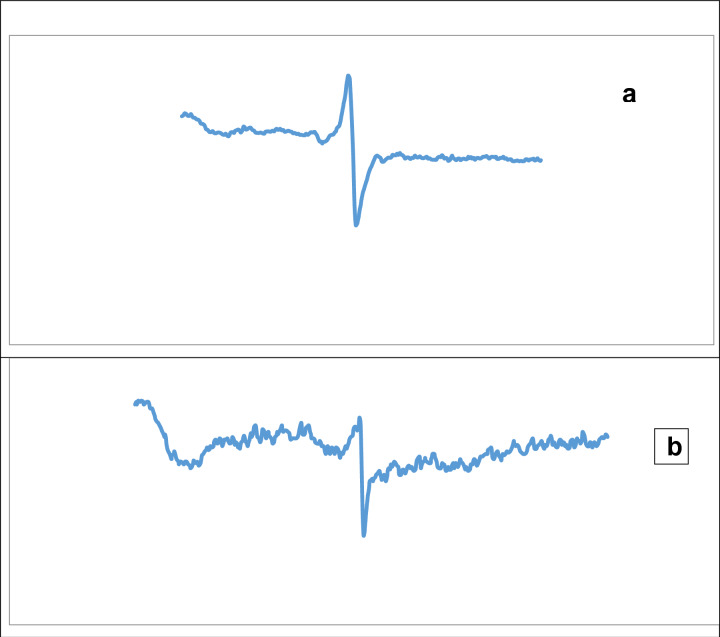
ESR spectra confirming the effects of SeNPs exposure on samples (**a**) ESR spectrum of negative control, (**b**) ESR spectrum of sample exposed to SeNPs.

*P. aeruginosa* was exposed to SeNPs during its growth along with 0.157 μg/ml, the signal of the free radical was then recorded. As shown in Fig. 14, ESR spectra increase in intensity under exposure condition 14(a) as compared to the control without nanoparticles (b).

**Figure 15:**
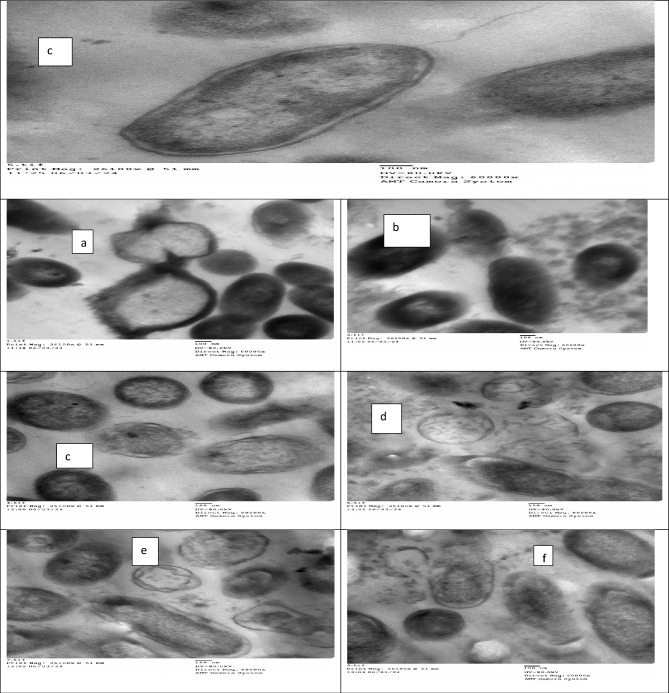
TEM of *P. aeruginosa* treated by SeNPs Scale Bar = 100 nm, 26,100 X. (**a**) Control and from (**b**–**f**) treated.

Ultrastructure studies by TEM reflect the presence of changes in *P. aeruginosa* which treated with Se NPs (Fig. 15) either in cells or on its flagella also. The treated *P. aeruginosa* cells at 0.157 µg/mL of SeNPs had higher cytoplasmic shrinkage with irregularities and vacuolization was observed. Cell wall deformation in shape, a leakage of cytoplasm in which the internal components of cells get out because of cell wall rupture, flagella cutting and accumulated, the cell membrane was detached from the cell wall, finally, cell collapsed and death. In contrast, control cells of *P. aeruginosa* were examined in identical intact cells, perfect shape, and clear healthy cell walls; including membranes and cytoplasm.

## Discussion

The novelty of this study is the synthesis of Se NPs using the gamma irradiation technique as a clean tool for polymerization, the reduction of selenium ions to selenium NPs without using reducing agent, and the preparation of (polyvinyl pyrrolidone (PVP)/Acetic Acid/glycerol/Se) without using chemical initiators or crosslinker. Consequently, the produced (PVP/Se NPs) is safe, clean, sterilized has no byproduct. The produced Se NPs can be used as effective anticancer and antimicrobial materials (which means 2*1 product).

Most of the nanoparticles that appear by HRTEM are separated from each other owing to the protection of the PVP network^28^.

Geoffrion et al.^1^ confirmed the sphericity of the SeNPs and their amorphous structure determined by electron diffraction, with a size of 32.3 ± 5.6 nm, 22 and 40–56 nm. Also, Al-Hakimi et al.^29^ prepared PVA/CMC/Se NPs films via the solution casting method. The Se NPs were characterized using a Transmission Electron Microscope (TEM), and the nanoparticles were nearly hollow spherical with a diameter range between 40 and 56 nm.

The HRTEM images (second run) of Se NPs show spherical and quasi-spherical patterns with an average particle size around 47.26 nm. Besides that, as seen in Fig. 3h, the polycrystalline structure of gamma radiation synthesized Selenium nanoparticles was evidenced by its correlating SAED assessment. The HR-TEM image also shows a well-defined inner surface of the ligament, with the d–widths for adjacent area adhesive fringes estimated to be 0.28 nm, which corresponds to the proportion of the (101) planes of t-Se plane of selenium nanoparticles suggesting the trigonal crystalline phase of these nanostructures.

From the literature, our results are acceptable with the work achieved by^30^. Also, Elakraa et al.^31^ reported the synthesis of selenium nanoparticles via gamma irradiation and the HRTEM investigations revealed that the shape of Se NPs is spherical.

As shown in Fig. 4b, the basic structure and purity of the synthesized Se NPs were determined using an EDX examination. The selenium element showed distinct absorption peaks in Se NPs at 1.40 keV and a small peak at 11.25 keV. The purity of the selenium element is confirmed by the EDX spectra, and the O and C peaks originate from PVP, which is employed to cap selenium nanoparticles. Table 2 shows that the relative elemental percentage of selenium is 2.75%. This indicates that after converting selenium ions to Se NPs, the surface area increased.

Figure 5 shows the elemental mapping of Se NPs. This image illustrates clearly that Se atoms are uniformly dispersed throughout the PVP network that C and O are connected to. The elemental mapping results show that gamma irradiation was a successful method for producing selenium nanoparticles capped with PVP. Additionally, it is an indication for reducing the selenium ions to selenium nanoparticles which are orderly homogenous as the surface area increases, too.

XRD results confirmed the formation of Se NPs and displayed the diffraction characteristics regarding 2θ at 22.7°(100), 27.6°, 30.4°, 31.8°, 39.2°, 41.3°, 48.3°, 49.7°, 56°, 58.5°, and 62°. In addition, there is a peak around 2θ = 20° which is attributed to PVP polymer. All the mentioned peaks were acceptable with the Joint Committee on Powder Diffraction Standards (JCPDS) of Se NPs with a standard card JCPDS File no 06-0362 and corresponding to (100), (101), (111), (110), (112), (202), (210)^32^. The obtained XRD data are appropriate with the results stated by^33^.

The gradual synthesis of Se-NPs nanoparticles utilizing gamma irradiation was evidently demonstrated by the generation of absorbance maxima at 280 and 570 nm. For 24 h, UV–Vis absorption spectrophotometry was thus applied to the material. Figure 7 displays the results. Because of the apparent lack of considerable movement or shift in the absorption peak, the nano-selenium particles showed good stability and did not undergo significant aggregation. Considering this, the preparation technique employing gamma irradiation to convert selenium ions into selenium nanoparticles that stabilize in the PVP network has proven to be successful. These results are similar to the results published by^34^.

Gautam et al.^35^ synthesized SeNPs were primarily confirmed by pink color when reduction of sodium selenosulphate. The change in absorption spectra was first carried out by UV spectroscopy in the range of 200–350 nm.

The characteristic peaks related to the functional groups of the polyvinylpyrrolidone (PVP); the peaks at approximately 3433 and 1368 cm^−1^ are attributed to the O–H stretching vibration The band at 1648 cm^−1^ is associated with the C=O stretching vibration, and the bands at 1427 cm^−1^ to the methylene group (CH_2_) bending vibration, while the peak at 2945 cm^−1^ is related to the asymmetric stretching vibration of the CH group^36^. In addition, the signals related to the C–N bond at 1276 and 1074 cm^−1^^37^.

The observed band at 2170.8 cm^−1^ belongs to C–C=–C–C=–CH, the noticed peak at 2024.96 cm^−1^ is assigned to C=C and C=N stretching^38^, also increasing the intensity of FT-IR peaks located at 1648 and 1042 cm^−1^ which indicates the chemical reaction between the added components PVP and selenium nanoparticles^39^. As noted in Fig. 4b, there are differences after the preparation of selenium nanoparticles using PVP solution combined with the additive’s acetic acid and glycerol; the displacement of the peak centered at 3433–3368 cm^−1^. Also, the intensity increased for the same peak. Moreover, the FTIR spectra showed differences in the intensity and broadness of the peaks due to the chemical interaction between Se NPs and the polymeric network, the other most significant differences are as follows; The observed band at 2170.8 cm^−1^ and the noticed peak at 2024.96 cm^−1^. Furthermore, it was found the decreasing intensity of FT-IR bands at 1434 and 1287 cm^−1^ and disappearing at the peak of 1368 cm^−1^ also. Furthermore, the displacement of the peak was found at 1074–1042 cm^−1^^40^.

Figure 9 displays the SeNPs’ Raman spectra. The primary peak, which is indicative of the amorphous phase in SeNPs, was detected at 254 cm^−1^. In particular, the A1 stretching Se-Se mode is linked to the band at 254 cm^−1^. Zhang and colleagues discovered comparable outcomes, revealing trigonal Se (t-Se) and monoclinic Se (m-Se) at 234 and 254 cm^−1^, respectively^41,42^.

The nano-Se inhibits cancer cell bioenergetics via glucose uptake slight blockage^43^ and its inhibitory concentration (IC50) was 50 μg/mL^44^.

In the past decade, selenium-containing nanomaterials have attracted much attention in the area of cancer therapy. Besides being used as anticancer drug carriers, they also showed anticancer activities based on the unique biological properties of selenium. In the form of selenocysteine, selenium is incorporated into many selenoproteins and exerts the ability to regulate the redox balance of the human body. Also, selenium shows antioxidant properties at low nutritional levels. However, it will turn into a pro-oxidant at elevated doses, which induces the production of ROS. Compared with healthy cells, cancer cells are characterized by higher levels of both ROS and reducing agents due to the accelerated glycolysis and pentose phosphate cycle^8,45^.

The dose of 100 μg/ml of Nanocomposite PVP/Se NPs-Chit was cytotoxic as confirmed by 5 and at lower concentrations, more than 80% of cells survived.

Gram-negative bacteria were more affected by selenium nanoparticles than gram-positive could be due to the gram-positive bacteria having a molecular network in the cell wall stronger than that in gram-negative bacteria, causing selenium ions to penetrate the cell walls of G−ve bacteria easier than that of G+ve bacteria^46^.

In general, the antimicrobial impact of nanoparticles associated with their structure/surface properties and charge type, thickness, and size in the nanoscale of these samples, the synergistic effect of Se NPs, besides the bacteria structural properties and the negative charge of bacteria surface. In other words, due to the high porosity of the cell wall and the most G -ve, the electrostatic attraction of the positively charged NPs to the surface of the cell wall can facilitate permeation, thus increasing the bactericidal effect of the samples^47,48^.

The known antibacterial mechanism of Se NPs is their adhesion to the bacterial surface and change of cell membrane integrity followed by the diffusion of small Se ions into the cells, these increase oxidative stress by generating high levels of intracellular reactive oxygen species (ROSs), which inhibit protein synthesis and cause DNA mutation, cell growth stops, and finally, cell death occurs^48,49^.

Menazea et al.^46^ found a dose-dependent inhibition of bacterial growth when exposed to different concentrations of Nanocomposite PVP/Se NPs, A 1 ppm concentration was enough to cause inhibition in bacteria proliferation, with a clear significance compared to smaller concentrations. Also, Nanocomposite PVP/Se NPs with a diameter of around 100 nm significantly inhibit the growth of *S. aureus* at a concentration as low as 7.8 μg/mL, furthermore, it is a known fact that the internalization of nanoparticles increases with the decrease in their size^5^.

From the literature, as compared to antibiotics, our MIC results are acceptable and more effective than the work achieved by, Silva et al.^50^. The antibacterial Gentamicin against *E. coli* 06, MIC was 25, for *P. aeruginosa* was 6.0, and for *S. aureus* 8.0 μg/mL. And with Norfloxacin, 5 for *E. coli*, 6.34 for *P. aeruginosa* and 161 μg/mL for *S. aureus*. And with Imipenem, 6.85 for *E. coli*, 2.5 for *P. aeruginosa*, and 4.5 μg/mL for *S. aureus.*

The results of MIC showed a dose-dependent inhibition of bacterial growth when exposed to different concentrations of PVP/Se NPs^1^.

Synthesized PVP/SeNPs showed MIC values around 2.35 when cultured with MDR-*E. coli*, while 4.45 ppm when PVP/Se NPs were used to inhibit the proliferation of *P. aeruginosa*, for *S. aureus* was 14.26, for *E. coli* was 12.77 ppm, as reported by^1^.

Also, other literature showed MIC values around 100 ppm when cultured with P. aeruginosa^51^. In comparison, MIC values of 125 ppm were found when PVP/Se NPs were used to inhibit the proliferation of both *S. epidermidis* and *S. aureus*^52^, and for *E. coli*, MIC values of 15 ppm were reported by^53^ when PVP/Se NPs used as antimicrobial agents, which all were lower or the same as in this study.

The lower MIC values calculated in the present work demonstrate that Se NPs synthesized by irradiation showed higher colloidal stability and are more efficient in killing bacteria than their counterparts synthesized by wet chemistry. These may be due to the naked surface of the Se NPs being directly in contact with the bacteria’s surface^1^.

The cells’ destruction action of PVP/SeNPs cleared by SEM depended on the combined actions of the nanocomposite constituents. Many reports principally attributed the antimicrobial actions to the interactions with microbial cells’ membranes and phospholipids, denaturation of wall protein, and distortion of membranes’ permeability^54^. Additionally, the strong electrostatic interactions between negatively charged cell membranes/walls and positively charged nanoparticles could increase the attachment to bacterial cells and their penetration and interactions with interior organelles of bacteria^55^. Accordingly, it could have additional probabilities for distorting bacterial cell walls/membranes and interfering with cells’ energetic components and functions to prohibit them, disrupt outer membranes’ synthesis and obstruct cells’ development, and induce severe walls’ lyses, and deformation^24,56^.

ROS is an initial source of nanomaterial toxicity that can be formed after exposure and inherent nanomaterials to elements. However, ROS's in situ identification and quantification have some obstacles, such as their interactions with repair systems and other factors, in which Electron Spin Resonance (ESR) is useful for detecting ROS formation^57^, the oxidative damage of cellular biomolecules, such as nucleic acids, and lipoprotein due to the reactive oxygen species (ROS)^58^.

Ultrastructure studies by^18,59^ treated *P. aeruginosa* with Se NPs at 31.2 µg/ml reflected the same changes when treated with Se NPs at 0.625 µg/ml in this study. The higher cytoplasmic shrinkage (or disappearance) with irregularities and ruptures of the cytoplasmic membrane, internal vacuolization, and complete deformation in shape appeared.

## Conclusion

Selenium is an essential micronutrient for all mammals, plays a role in maintaining human physiological functions, and affects the production and reproductive properties. Providing an adequate supply of selenium to the diet prevents health problems from deficiency. Due to its high bioavailability, low toxicity, and affordability, selenium in its nanoform appears to be the most appropriate for supplementation. In recent years, multidrug-resistant (MDR) bacteria have increased rapidly and represented a threat to human health. This problem has created an urgent need to identify alternative methods for its treatment. Accordingly, selenium nanoparticles (Se NPs) were successfully prepared via gamma irradiation using PVP as a stabilizing agent. The formation of Se NPs confirmed using HRTEM and UV–vis spectroscopy. In this study, the prepared Nanocomposite PVP/(Se NPs) based on Se NPs doped in a PVP network demonstrate antimicrobial and antitumor activity. The cytotoxicity of Se NPs toward MRC-5 cells was determined, and a significant difference was noticed on the MRC-5 cell line in the same concentration interval as antimicrobial testing. Moreover, the antitumor activity of the synthesized Nanocomposite complex was determined using a colorimetric MTT assay that showed variation in inhibitory activity to the five tested carcinoma cell lines in a concentration-dependent manner. In addition, the antimicrobial activity studied on five bacterial strains (*S. aureus*, *B. cereus, K. pneumoniae*, *E. coli*, and *P. aeruginosa*), in which PVP/Se NPs nanocomposites exhibit excellent antibacterial activity for positive and negative strains, it was higher activity against Gram-negative bacteria in which *K.* pneumoniae and *P. aeruginosa* was the most sensitive strain to SeNPs. MIC reaches to 0.313 μg/ml. The antimicrobial was examined by the broth macro-dilution method. The qualitative antibacterial activity results showed an increase in the diameter zone with increasing nanocomposite concentration and the highest zone was 36.33 ± 3.05 mm. The complete rupture of the cells and cutting of their flagella, which were clarified by TEM appeared because of the free radicals which were confirmed by ESR and confirmed their cytotoxicity.

In the future, Se NPs will be prepared within the network of biodegradable polymers such as chitosan, Gum Arabic, etc. via ionizing radiation for the potential application of Se NPs in agriculture. Se NPs could mitigate biotic and abiotic stresses in plants. Se NPs could promote seed germination and plant growth. Se NPs could improve Se contents and nutritional values in crops.

## Data Availability

Data will be made available on request from abir2partila@yahoo.com.
